# Editorial: Innovations in uniportal lung resection

**DOI:** 10.3389/fsurg.2026.2028383

**Published:** 2026-10-28

**Authors:** Yojiro Yutaka, Calvin Sze Hang Ng

**Affiliations:** 1 Department of Thoracic Surgery, Kurashiki Central Hospital, Okayama, Japan; 2 Division of Cardiothoracic Surgery, The Chinese University of Hong Kong, Hong Kong, Hong Kong SAR, China

**Keywords:** lung resection, minimally invasive thoracic surgery, reduced-port surgery, single-port robotic surgery, surgical education, uniportal VATS

Minimally invasive thoracic surgery has undergone remarkable evolution over recent decades, progressing from conventional multiportal video-assisted thoracoscopic surgery (VATS) to uniportal VATS (uVATS) and robot-assisted thoracic surgery (RATS). Uniportal VATS has become an established approach for anatomical lung resection, supported by continuous innovations in surgical instruments, visualization, and operative techniques (1). More recently, reduced-port and single-port robotic approaches have further expanded the possibilities of minimally invasive lung surgery. However, the true value of these innovations should not be measured solely by the number or size of surgical incisions. Rather, their clinical significance lies in achieving safe and oncologically sound resections while minimizing surgical trauma, preserving pulmonary function, and improving patient recovery.

This Research Topic, Innovations in Uniportal Lung Resection, brings together nine articles addressing patient-centered outcomes, technical refinements, robotic innovations, and the expanding role of minimally invasive thoracic surgery. Collectively, these contributions highlight both the progress achieved and the important challenges that remain.

One of the principal motivations for uniportal surgery is the potential reduction in postoperative pain and chest wall trauma. Kanani et al. conducted a systematic review and meta-analysis of 19 studies involving 2,544 patients, demonstrating lower postoperative pain scores following uVATS compared with multiportal VATS. Although heterogeneity across studies warrants consideration, these findings support the potential clinical advantages of minimizing intercostal access.

Another essential aspect of minimally invasive surgery is the preservation of functional lung parenchyma. Zhang et al. introduced the CONSERVO technique, a conformal sublobar electroresection approach designed to reduce unnecessary parenchymal loss during wedge resection. Their volumetric analysis demonstrated greater parenchymal preservation than conventional stapler-based resection, although operative time was longer. This study highlights an important principle: minimizing the amount of healthy lung tissue removed may be as clinically relevant as minimizing chest wall trauma. Similarly, Zhang et al. evaluated the HilumDirect uVATS technique for small cell lung cancer, illustrating continued efforts to refine hilar dissection and lymph node assessment.

The emergence of single-port robotic platforms represents another important development. Lee et al. evaluated anatomical segmentectomy using the da Vinci SP system and reported favorable short-term perioperative outcomes. Likewise, Kim and Yun demonstrated the feasibility of transitioning from multiport to uniport RATS, with encouraging perioperative outcomes. These studies suggest that uniportal access and robotic technology may represent complementary rather than competing innovations. Nevertheless, their widespread adoption requires careful consideration of surgical ergonomics, instrument interference, learning curves, and procedural safety.

The expanding indications for minimally invasive surgery are equally important. Yongue et al. reported encouraging perioperative outcomes following robotic anatomical lung resection after neoadjuvant therapy for non-small cell lung cancer. Similarly, Asban et al. described their experience with robotic pneumonectomy, illustrating the feasibility of complex resections in highly experienced centers. Although these investigations do not specifically establish the benefits of uniportal access, they demonstrate how advances in visualization, instrumentation, and surgical techniques may extend minimally invasive approaches to increasingly challenging cases. Furthermore, Hosseinzadeh et al. reported single-port subxiphoid robotic excision of ectopic mediastinal parathyroid adenomas, highlighting the potential applications of single-port technology beyond pulmonary resection.

Importantly, technological innovation must be accompanied by systematic surgical education. The transition from multiportal to uniportal surgery involves fundamental changes in instrument manipulation, visualization, and coordination between the surgeon and surgical assistants. In our previous study, we evaluated the safe introduction of uVATS in an academic center, focusing on the learning curve, stepwise surgical training, and structured team-based education (2). Our experience highlights the importance of establishing a standardized educational framework that enables surgeons to acquire new technical skills while maintaining procedural safety. Such considerations are increasingly relevant as thoracic surgery evolves toward diversified approaches, including uVATS, reduced-port RATS, and single-port robotic surgery.

Furthermore, the integration of surgical techniques with multidisciplinary technologies will be essential for future progress. In their review, Chang et al. discussed the convergence of uniportal surgery with advanced imaging, bronchoscopic localization, neoadjuvant treatment, and emerging hybrid therapeutic approaches. These developments extend the concept of innovation beyond surgical access and highlight the importance of coordinating diagnostic, systemic, and local treatments within a comprehensive patient-centered strategy. Such integration reflects the broader evolution of minimally invasive thoracic surgery, as discussed in our previous Editorial (3).

Despite these promising developments, several challenges remain. Much of the available evidence is derived from retrospective studies conducted at experienced institutions, and long-term oncological outcomes remain insufficiently established for newer techniques. Standardized training, objective assessment of surgical proficiency, prospective comparative studies, and economic evaluations will be essential to establish the safety, reproducibility, and clinical benefits of these evolving approaches.

Ultimately, the future of minimally invasive thoracic surgery should be guided by a patient-centered philosophy. Uniportal VATS, reduced-port RATS, and single-port robotic surgery should be regarded as complementary options rather than competing techniques, with the optimal approach selected according to individual patient characteristics and surgical complexity. Continued innovation should prioritize oncological completeness, procedural safety, preservation of pulmonary function, and meaningful improvements in patient recovery. We hope that the contributions to this Research Topic will stimulate further innovation, critical evaluation, and international collaboration toward these shared goals.
